# Valuing health‐related quality of life: An EQ‐5D‐5L value set for England

**DOI:** 10.1002/hec.3564

**Published:** 2017-08-22

**Authors:** Nancy J. Devlin, Koonal K. Shah, Yan Feng, Brendan Mulhern, Ben van Hout

**Affiliations:** ^1^ Office of Health Economics London UK; ^2^ School of Health and Related Research University of Sheffield Sheffield UK; ^3^ Centre for Health Economics Research and Evaluation University of Technology Sydney Sydney NSW Australia

**Keywords:** EQ‐5D‐5L, NICE, PROMs, quality of life, stated preferences

## Abstract

A new version of the EQ‐5D, the EQ‐5D‐5L, is available. The aim of this study is to produce a value set to support use of EQ‐5D‐5L data in decision‐making.

The study design followed an international research protocol. Randomly selected members of the English general public completed 10 time trade‐off and 7 discrete choice experiment tasks in face‐to‐face interviews.

A 20‐parameter hybrid model was used to combine time trade‐off and discrete choice experiment data to generate values for the 3,125 EQ‐5D‐5L health states.

Valuation data are available for 996 respondents. Face validity of the data has been demonstrated, with more severe health states generally given lower values. Problems with pain/discomfort and anxiety/depression received the greatest weight. Compared to the existing EQ‐5D‐3L value set, there are considerably fewer “worse than dead” states (5.1%, compared with over one third), and the minimum value is higher. Values range from −0.285 (extreme problems on all dimensions) to 0.950 (for health states 11211 and 21111).

Results have important implications for users of the EQ‐5D‐5L both in England and internationally. Quality‐adjusted life year gains from interventions seeking to improve very poor health may be smaller using this value set and may previously have been overestimated.

## 1 INTRODUCTION

Health care decisions are made under uncertainty, whereby any decision may have a range of different outcomes. To make the “best” decision, potential outcomes need ordering and valuing. Such decisions are made both at the individual level, such as choosing the optimal treatment for a patient, and at the national level, such as choosing how to allocate resources between treatments for different patient groups and across different health conditions.

Clinical decisions often affect patients' health‐related quality of life (HRQL). Evidence on patients' HRQL can be obtained using patient‐reported outcome (PRO) measures. These may be condition specific or generic (see Fayers and Machin, 2016, and Longworth et al., 2014, for further information). Condition‐specific PROs focus on specific health problems and aim to provide detailed information about the impacts of the condition, disregarding problems that are atypical for the condition. Generic PROs aim to cover a more general spectrum of health problems and are designed to be applicable for any health condition. They can capture comorbidities and allow comparisons with population norms. Evidence obtained from generic measures can be used to compare both the impact of health problems and the benefits offered by treatments across different patient populations and disease areas. This makes these data particularly useful for informing decisions made by health care professionals, commissioners, regulators, health technology assessment bodies (such as the National Institute for Health and Care Excellence [NICE]), payers, and budget holders.

The EQ‐5D is the most widely used generic PRO questionnaire internationally (Devlin & Brooks, 2017; Richardson, McKie, & Bariola, 2014). It is the instrument recommended by NICE for evidence submitted to its technology appraisal process (NICE, 2013). It has also proved useful in population health surveys and in the English National Health Service (NHS) patient‐reported outcome measures programme (Devlin & Appleby, 2010). The EQ‐5D asks patients to indicate whether they have no, some, or extreme problems on each of five dimensions of health: mobility, self‐care, usual activities, pain/discomfort, and anxiety/depression.

**Figure 1:**
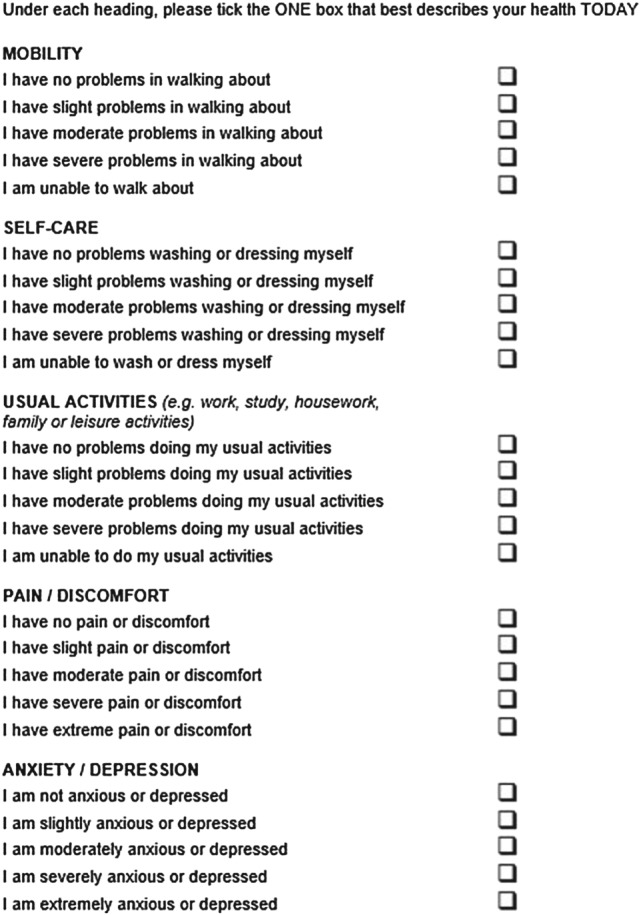
The EQ‐5D‐5L descriptive system

The EQ‐5D is a valid and reliable measure in many disease areas (Janssen, Lubetkin, Sekhobo, & Pickard, 2011; Pickard, Wilke, Lin, & Lloyd, 2007; Wailoo, David, & Tosh, 2010). However, there have been concerns that three response options for each dimension may not adequately capture milder health problems experienced by patients, and smaller changes between different health states. A new version of the instrument, the EQ‐5D‐5L, was developed to improve sensitivity and to standardise the language used across dimensions (Herdman et al., 2011). The EQ‐5D‐5L comprises the same five dimensions, but increases the available response options (levels) from three to five (no, slight, moderate, severe, and extreme problems/unable to)—see Figure 1. The five dimensions and five levels of the EQ‐5D‐5L describe 3,125 (5^5^) unique health states, compared to the 243 (3^5^) described by the EQ‐5D.

The EQ‐5D‐5L is rapidly being incorporated into routine data collection in clinical settings, clinical trials and population health surveys, such as the GP patient survey (NHS, 2016) and the Health Survey for England (Fuller, Mindell, & Prior, 2016). It is also used in local initiatives. For example, the Cambridgeshire Community Services NHS Trust collects EQ‐5D‐5L data to evaluate outcomes from rehabilitation services (Cambridgeshire Community Services NHS Trust, 2013–2014). Its design accounts for the need for a direct link between the measurement and valuation of health, whereby every health state that patients might report on the EQ‐5D‐5L instrument can be summarised by a single value. In order to be used in the calculation of quality‐adjusted life years (QALYs; a metric used in cost‐utility analysis that combines survival and HRQL), these values need to summarise how good or bad each health state is on a scale anchored at 1 (*full health*) and 0 (*a state equivalent to dead*). Values less than 0 represent health states considered to be or modelled as worse than dead. The values are based on the views of the general public who are asked to imagine living in various health states and to respond to a series of structured questions designed to find out the importance to them of different aspects of health. This approach follows the requirements of NICE (2013) and similar organisations (e.g., Australia's Pharmaceutical Benefits Advisory Committee, 2016, and the Canadian Agency for Drugs and Technologies in Health, 2006) for the use of EQ‐5D‐5L data in decision‐making and reflects a belief that it is the views of the general public—as taxpayers and potential users of health care—that should count, rather than simply those of patients (Neumann, Ganiats, Russell, Sanders, & Siegel, 2017).

At the time of writing, 25 value sets for the (three‐level) EQ‐5D‐3L are available (Devlin & Brooks, 2017), a number of which are summarised by Szende, Oppe, and Devlin (2007). The current U.K. EQ‐5D‐3L value set (Dolan, 1997)—which was developed based on data collected in the early 1990s—has values that range from 1 for no problems on any dimension to −0.594 for the worst health state (Level 3 problems on each dimension). A number of limitations have been noted with that value set. Among these are that approximately a third of health states described by the EQ‐5D‐3L were assigned negative values, meaning those health states are valued as being worse than dead. Although this may reflect genuine preferences, it is worth noting that the U.K. EQ‐5D‐3L values are rather unique in this respect: all other countries have fewer worse than dead health states and higher minimum values. Another generic measure with a U.K. value set—the SF‐6D (Brazier, Roberts, & Deverill, 2002)—includes no health states valued as worse than dead. Additionally, although also common to other countries, any change in health away from full health to “some” problems, on any aspect of health, results in a large fall in the overall value (of at least 0.12, meaning that the highest value for a health state describing at least some health problems is 0.88) on the 0 to 1 scale. The issues with the current EQ‐5D‐3L value set—particularly those regarding the larger number of health states modelled as worse than dead and the large differences in utility between adjacent levels—may be linked to features of the instrument (such as the labels used to describe different severity levels), the methods used to elicit preferences, and the modelling process used to derive value sets. Issues with the modelling used in the U.K. EQ‐5D‐3L value set study have been raised in the literature (Hernández Alava, Wailoo, & Ara, 2012). For example, it has been argued that the application of linear regression methods for modelling skewed data, and the use of an N3 term (see below), extended the range of the utility scale. It also contributed to the distinctive two‐group distribution often noted when applying the value set to patients' data (Parkin, Devlin, & Feng, 2016). The characteristics of the EQ‐5D‐3L value set may have important implications for decisions being made in the NHS. For example, NICE estimates of QALY gains from new treatments that restore patients with health problems to a state of full health may be biased upwards, because they will be improving from a relatively low baseline.

To date, there have been no values specific to the (five‐level) EQ‐5D‐5L in England available to summarise patients' data. Research has established the relationship between patients' self‐reported health on the EQ‐5D‐3L and on the EQ‐5D‐5L, enabling EQ‐5D‐5L data collected from patients to be summarised using the EQ‐5D‐3L value set via a “mapping algorithm” (van Hout et al., 2012). This provides an interim means of scoring EQ‐5D‐5L data, but perpetuates the limitations of the EQ‐5D‐3L value set (Devlin, Tsuchiya, Buckingham, & Tilling, 2011; Devlin et al., 2013; Tilling, Devlin, Tsuchiya, & Buckingham, 2010).

The aim of this study is to produce a value set for the EQ‐5D‐5L that can be used to support decision‐making in the English NHS. The results will have international impact given that decisions made by NICE influence health technology assessment decisions around the world (Hernandez‐Villafuerte, Garau, & Devlin, 2014). The study is relevant to clinicians collecting PRO data from patients and to those using PRO data in health care decisions. It demonstrates the relative importance placed on different types of health problems by people in England—and how that should be reflected in priority setting.

## 2 METHODS

The research design and data collection followed a protocol developed by the EuroQol Group, a not‐for‐profit international network of multidisciplinary researchers. The protocol was informed by an extensive programme of methodological research investigating methods for valuing EQ‐5D‐5L health states (Oppe, Devlin, van Hout, Krabbe, & de Charro, 2014). Our study was one of the first to use the protocol, and comparable studies in other countries have either recently concluded or are currently in progress.

### 2.1 Methods of eliciting preferences

The study used the EuroQol Valuation Technology (EQ‐VT) software, developed specifically for EQ‐5D‐5L value set studies and administered using computer‐assisted personal interviews. Two stated preference methods were used to elicit preferences: time trade‐off (TTO), an approach used in previous EQ‐5D valuation studies (Oppe et al., 2014) and accepted by NICE as a “choice‐based” approach (NICE, 2013); and the discrete choice experiment (DCE), an approach that is increasingly used to assess preferences for health states because of the relative simplicity of the tasks (Ryan, Gerard, & Amaya‐Amaya, 2008). The two methods generate different and complementary preference data. TTO elicits a value for each state with 1 and 0 defined as anchor points, whereas DCE generates binary data that allow for the derivation of a scale of nonanchored relative values.

Each interview consisted of the following tasks (in order): self‐reported health using EQ‐5D‐5L, self‐reported health on a 0–100 visual analogue scale; basic background questions (common to all value set studies administered via EQ‐VT); a practice TTO task (involving the valuation of a simple health state that described requiring the use of a wheelchair); 10 TTO tasks; structured feedback questions regarding the TTO tasks; seven DCE tasks; structured DCE feedback questions; an (optional) open‐ended comment box; and further England‐specific background questions.

**Figure 2:**
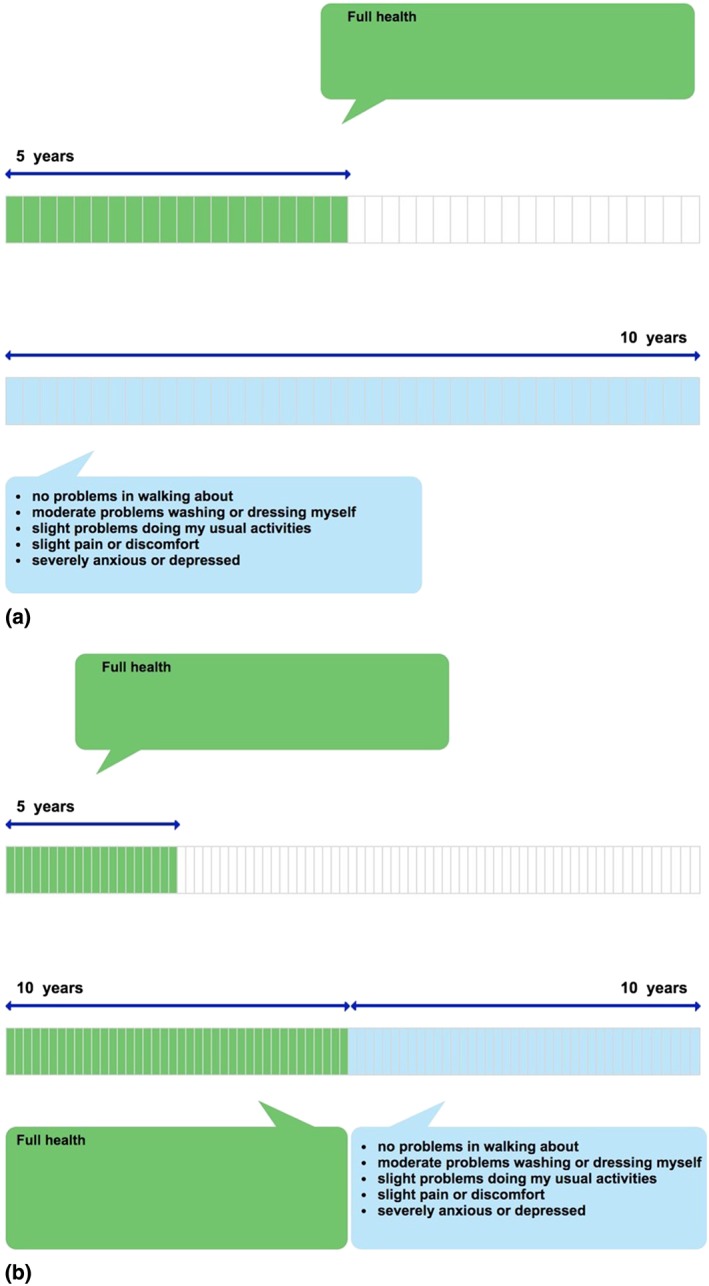
(a) Example of time trade‐off valuation of health states better than dead (i.e., values ≥ 0). (b) Example of time trade‐off valuation of health states worse than dead (i.e., values ≤ 0) [Colour figure can be viewed at wileyonlinelibrary.com]

In the TTO tasks, a composite approach was used that involved starting with the “conventional” TTO (Brazier, Ratcliffe, Salomon, & Tsuchiya, 2017) for all health states and shifting to a “lead time” TTO when participants indicated that they considered the health state to be worse than dead (Devlin et al., 2011; Devlin et al., 2013; Robinson & Spencer, 2006). The composite TTO approach is illustrated in Figure 2a,b. Evidence supporting this approach is reported by Janssen, Oppe, Versteegh, and Stolk (2013). Figure 2a illustrates the TTO task for health states better than dead (i.e., those with a value between 0 and 1). The participant is asked to imagine living for 10 years from today in a given EQ‐5D‐5L state, followed by death (Life B). The participant's value for that health state is then derived by identifying, using an iterative process combining bisection and upward/downward titration approaches (Oppe, Rand‐Hendriksen, Shah, Ramos‐Goñi, & Luo, 2016), the number of years in full health between 0 and 10 (Life A) they consider equivalent to that. The more severe the health state described in Life B, the more years of full health the participant is assumed to be willing to give up in Life A to avoid Life B. For very poor health states, all of the time in Life A may be traded off, indicating that the value for the state is less than or equal to 0. Where this occurs, additional time in full health (lead time) is added to both Lives A and B—see Figure 2b. This allows participants to trade‐off more time, reflecting how much worse than dead they consider the health state to be (within the boundaries of the scale imposed by the task; Devlin et al., 2013).

The variant of lead time TTO used in this study involved a 20‐year time frame (10 years of lead time followed by 10 years in the health state under evaluation), yielding a minimum value of −1. No additional trade‐off questions were asked of those who exhausted their lead time (Devlin et al., 2013). The iterative procedure used to seek the point of indifference was based on an adaptation of that used in the U.K. EQ‐5D value set study (Dolan, 1997). Further details about EQ‐VT and the iterative process used in the TTO tasks are provided by Oppe et al. (2014).

Each TTO task ends when the participant indicates that they consider Lives A and B to be “about the same.” At this point of indifference, the implied value for health states better than dead is calculated by dividing the total number of years in Life A (*t*) by 10 (the total number of years in Life B). This can be expressed as *V* = *t*/10, where *V* is the health state value. For example, at the point of indifference shown in Figure 2a, the health state value would be 5/10 = 0.5. The implied value for health states worse than dead is calculated by subtracting 10 (the number of years of lead time) from the total number of years in Life A, then dividing by 10 (the total number of years in Life B minus the number of years of lead time). This can be expressed as *V* = (*t* − 10)/10. The point of indifference shown in Figure 2b would suggest a value of (5–10)/10 = −0.5. The maximum value is 1, achieved when the participant considers 10 years in the health state to be as good as 10 years in full health. A value of 0 is given when the participant considers the health state to be no better and no worse than dead. The minimum score of −1 (where all of the lead time is exhausted) is given when the participant considers the prospect of living for 10 years in full health followed by 10 years in the health state to be worse than or equivalent to a life lasting 0 years (i.e., dying now).

**Figure 3:**
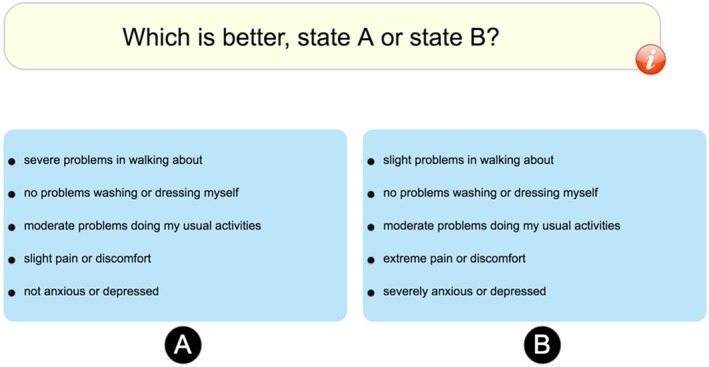
Example discrete choice experiment task [Colour figure can be viewed at wileyonlinelibrary.com]

In each DCE task (Figure 3), participants were presented with a pair of health states (labelled A and B), with no reference to the duration of the states, and asked to indicate which they considered to be “better” by clicking the appropriate button. No indifference option was included.

### 2.2 Study design

The TTO design included 86 states. Six health states—the worst state (55555) and the five mildest states (21111, 12111, 11211, 11121, and 11112) were selected *a priori*. The mild health states were included so that direct observations of these would ensure that we could statistically distinguish minor problems from full health. These states were supplemented with 80 states selected using the design strategy described in Oppe and van Hout (2010). This design was generated by randomly selecting 80 health states from the full fractional design minus the six health states noted above. An expected health state value was assigned to each of the 86 states in the design on the basis of priors. Subsequently, a regression model was estimated on the dataset to calculate predicted values for each health state. The strategy was “looped” until a design was identified with small differences between prior and predicted values. The 80 states were distributed over 10 blocks in such a way that the full utility range would be covered within a block and all blocks would have the same mean utility. In the final design, each block included eight health states unique to that block, plus state 55555 and one of the mild health states.

The DCE experimental design included 196 pairs randomly divided into 28 blocks identified using a Bayesian design. The blocks were balanced in terms of their severity, as assessed by the level sum scores of the health states (see below). None of the pairs in the DCE design included a health state that logically dominated the other (i.e., at least as good on all five dimensions). Participants were randomly assigned to one of the 10 blocks of TTO tasks and to one of the 28 blocks of DCE tasks. Block assignment, question order, and (in DCE) the left–right position of health states were all randomised.

The EQ‐5D‐5L valuation protocol (Oppe et al., 2014) suggests a target sample size of 1,000 participants. A previous study (Janssen et al., 2013) found that 100 observations per health state resulted in a small standard error of mean health state values between 0.01 (for mild states) and 0.06 (for poor states), which was considered sufficient. The number of TTO tasks per participant was set to 10, resulting in a sample size of 1,000.

### 2.3 Data collection

Sample recruitment and interviewing was carried out by the market research company Ipsos MORI. A sample of 2,220 addresses from 66 primary sampling units across England was randomly selected based on postcode sectors using the Post Office small user Postcode Address File. This includes all private residential accommodation in England (communal establishments, such as prisons and care homes, were excluded). Thirty‐seven addresses were selected systematically from an ordered list of all addresses within each sampling unit, ensuring that addresses were spread evenly across it. Interviewers sent an advance letter and information sheet to each selected address, together with an unconditional incentive of six first‐class stamps. In each selected dwelling unit, all individuals aged 18 years and over were listed in alphabetical order by first name, and one was selected randomly using a selection grid with no substitutes permitted.

If the selected individual gave their informed consent to take part, they were interviewed in their own home. The participant was in control of the computer (laptop) throughout the tasks, with the interviewer guiding them through each step, following a script. The one‐to‐one setting allowed interviewers to provide detailed instruction and feedback where appropriate (Shah, Lloyd, Oppe, & Devlin, 2013).

Forty‐eight interviewers were used, all of whom attended a full day briefing in which they were given intensive training on the methodology and study procedures by the research team. Interim data were monitored at the interviewer level, at least weekly. If an interviewer was found to be generating unusual or poor quality data (defined using criteria based on expected data characteristics, given previous research (e.g., Shah et al., 2013), such as minimum task completion times), they were given additional training. No data were removed at this stage.

### 2.4 Piloting

The main study was preceded by a small pilot study (*n* = 49), undertaken in August 2012. The pilot sample was recruited using quotas on age, gender, and working status rather than using the systematic approach described above. The aims of the pilot were to test for technical issues with EQ‐VT, to test Ipsos MORI's procedures and methods of encouraging participation, to seek interviewers' feedback on the preliminary script and other materials, to seek participants' feedback on the interview, to examine basic properties of the data generated, and to identify ways to improve the interviewer training process. The pilot was completed successfully, with no issues with EQ‐VT reported by participants or interviewers. Several improvements were made as a result of the feedback received during piloting. These included refinement of the interviewer script and improvements to the interviewer training process.

### 2.5 Methods of analysis

For both TTO and DCE, a range of descriptive analyses were conducted to assess data characteristics. For TTO, this included examining the overall distribution of values and correlating average values for each health state with its level sum score (a proxy for severity; e.g., the worst health state 55555 has a score of 5 + 5 + 5 + 5 + 5 = 25). Following the approach of Janssen et al. (2013), face validity of the TTO data was assessed by checking for the expected negative relationship between level sum score and average observed value. Face validity of the DCE data was assessed by comparing the proportion of participants choosing health state A with the difference in level sum scores between A and B, with the implicit assumption that health states with lower level sum scores would be more likely to be chosen overall.

To generate the modelling dataset, we tested a range of possible exclusion criteria, reflecting alternative judgements that might be made about the quality of the data. We wished to minimise exclusions but sought to omit data that were clearly implausible. The final rules for the TTO data were to exclude (a) participants who gave all 10 health states the same value (all health states cannot plausibly be given the same value given the severity range in each block) and (b) participants who gave the worst state, 55555, a value that was no lower than the value they gave to the mildest health state in their block. Both suggest either misunderstanding or lack of engagement.

No DCE data were excluded.

The EuroQol Group does not have a standard modelling protocol for the analysis of EQ‐5D‐5L valuation data, as different methods may be required to reflect the fact that the data characteristics are likely to vary across countries. The modelling methods in this study therefore explicitly addressed the observed characteristics of the data (Feng, Devlin, Shah, Mulhern, & van Hout, 2017). First, the minimum TTO value is bounded at −1 by design, so we allowed for the possibility of values lower than that using survival analysis approaches for treating censored data. Second, the maximum TTO value is 1, again by design, so although there is an error distribution around observed values, that distribution is necessarily asymmetric at 1, biasing the values for mild health states downwards. Thus, the values at 1 were also considered to be censored. Third, there were some participants who used 0 as the minimum value for more than one health state (almost always including health state 55555). This suggests that these participants were averse to giving negative TTO values. Those values were considered to be censored at zero. Furthermore, some participants gave health state 55555 a value of 0 whilst giving multiple other health states a negative value. This in an example of a “logical inconsistency,” because 55555 is dominated by all other health states. Those values were censored at 0. See Feng et al. (2017) for further details.

Further, we observed that participants more often disagree about the value of health states that are further away from full health, that is, the variance of TTO values increases for worse health states. Based on examinations of visual representations of the valuation data at the individual participant level to identify common patterns, it was apparent that different groups of participants differed in their use of the scale, resulting in substantially different slopes (i.e., the relationships between disutility and health state severity). This could fundamentally be driven by the heterogeneity of participants in their views about death. The effect of heterogeneity was explored using models that introduced a parameter for the scale of disutility in health, which may differ between participants. The scale of disutility in health was assumed to follow a multinomial distribution with probability density on a number of latent groups. Each of the latent groups has its mean and variance for the distribution of the scale. In our analysis, we assessed the fit of models with two to 10 latent groups using the deviance information criterion (DIC). We also accounted for heteroskedasticity within each latent group in the model. This was achieved by allowing variance for participants in different age groups to be different within a latent group.

Models were estimated with different degrees of freedom. The most restrictive model gives different weights to the five dimensions and assumes equal distances between levels. The second most restrictive adds different values for the levels with a distinction between “extreme” and “unable to” (the former is the Level 5 label used for the pain/discomfort and anxiety/depression dimensions; the latter is the Level 5 label used for the mobility, self‐care, and usual activities dimensions). The least restrictive model has a parameter for each decrement or step away from “no problems” (Level 1) on each dimension, thereby estimating 20 parameters (4 levels × 5 dimensions). Within these specifications, a range of alternative models was tested to capture the possibility of interaction effects between dimensions and levels.

Twenty parameter models that involved TTO data showed logical inconsistencies in some dimensions. In the model used to produce the EQ‐5D‐5L value set for England, we applied restrictions to the parameters. Specifically, the Level 2 parameters were estimated first, and parameters for subsequent levels were estimated by adding quadratic terms (which can be nonnegative only, thereby ensuring that moving to worse levels of problems always resulted in an increase in disutility).

Models were estimated separately for the TTO and DCE data and then using a hybrid modelling approach with a Bayesian regression model to combine the TTO and DCE data together (Ramos‐Goñi, Pinto‐Prades, Cabasés, & Rivero‐Arias, 2014; Rowen, Brazier, & van Hout, 2014).

The sociodemographic composition of the sample was checked for representativeness against the general population. The modelling approach adjusted for the age distribution of our sample to reflect the age distribution of the general population in England. Models were estimated on data specific to selected sociodemographic groups (e.g., male participants vs. female participants) to check for any systematic differences.

All analyses were conducted in R3.2.0 and Winbugs 14. The operating system for producing all results was Windows 10 Version 1607 for x64‐based systems. The results were produced on 10th January 2017.

The methods and analyses reported in this paper comply with the CREATE guidelines for reporting valuation studies of multiattribute utility‐based instruments (Xie et al., 2015). The modelling methods are described in greater detail in an accompanying paper by Feng et al. (2017).

## 3 RESULTS

### 3.1 Sample

The interviews were conducted between November 2012 and May 2013. Of the individuals invited to take part in the study, 996 completed the valuation questionnaire, comprising TTO tasks, DCE tasks, and basic background questions, in full (response rate = 47.7%). In accordance with the ethical approval for this study, participants who did not complete the valuation questionnaire in full were excluded from the analysis; hence, there are no missing TTO and DCE responses in our dataset. Full background data were collected for 985 of the 996 participants (98.9%). Table 1 shows that, compared to the general population, the sample includes a larger proportion of those aged over 75 and retired individuals and a smaller proportion of younger individuals and males (Office for National Statistics, 2011). The sample also includes a relatively large proportion of individuals with health problems (approximately 14% higher than the general population).

**Table 1:** Background characteristics of the sample

	All participants (*n* = 996)^a^	After exclusions (*n* = 912)^a^	General population^b^
	*N* (%)	*N* (%)	%
Age			
18–29	113 (11.3)	105 (11.5)	20.7
30–44	298 (29.9)	270 (29.6)	26.3
45–59	250 (25.1)	227 (24.9)	24.7
60–74	207 (20.8)	191 (20.9)	18.5
75+	128 (12.9)	119 (13.0)	9.9
Gender			
Male	405 (40.7)	372 (40.8)	49.2
Female	591 (59.3)	540 (59.2)	50.8
Economic activity			
Employed or self‐employed	504 (51.2)	463 (50.8)	59.4
Retired	278 (28.2)	256 (28.1)	13.1
Student	20 (2.0)	19 (2.1)	8.8
Looking after home or family	83 (8.4)	73 (8.0)	4.2
Long‐term sick or disabled	48 (4.9)	42 (4.6)	3.9
Other/none of the above	52 (5.3)	47 (5.2)	10.6
Marital status			
Never married	238 (24.2)	225 (24.7)	34.6
Married	466 (47.3)	434 (47.6)	46.6
Same‐sex civil partnership	2 (0.2)	2 (0.2)	0.2
Separated^c^	37 (3.8)	32 (3.5)	2.7
Divorced	131 (13.3)	119 (13.0)	9.0
Widowed^d^	107 (10.9)	99 (10.9)	6.9
Prefer not to say	4 (0.4)	1 (0.1)	N/A
Religion			
Christian	636 (64.6)	575 (63.9)	59.4
Any other religion	60 (6.1)	53 (5.9)	8.7
No religion	281 (28.5)	266 (29.6)	24.7
Religion not stated	8 (0.8)	6 (0.7)	7.2
Ethnicity			
White	900 (91.4)	832 (92.4)	85.4
Any other ethnic group	82 (8.3)	67 (7.4)	14.6
Prefer not to say	3 (0.3)	1 (0.1)	N/A
Day‐to‐day limitations due to health problem or disability			
Limited a lot	111 (11.3)	95 (10.6)	5.6^e^
Limited a little	158 (16.0)	144 (16.0)	7.1^e^
Not limited	716 (72.7)	661 (73.4)	87.3^e^
Education			
Degree	211 (21.4)	201 (22.3)	N/A
No degree	774 (78.6)	699 (77.7)	
Main language spoken			
English	920 (93.4)	847 (94.1)	N/A
Any other language	65 (6.6)	53 (5.9)	
Responsibility for children			
Yes	350 (35.5)	314 (34.9)	N/A
No	635 (64.5)	586 (65.1)	
Experience of serious illness			
In self	330 (33.1)	297 (32.6)	N/A
In family	692 (69.5)	636 (69.7)	
In caring for others	416 (41.8)	385 (42.2)	
Self‐rated health using EQ‐5D‐5L			
11111	474 (47.6)	437 (47.9)	N/A
Any other health state	522 (52.4)	475 (52.1)	
Self‐rated health using EQ‐VAS			
<80	334 (33.5)	298 (32.7)	N/A
80–89	256 (25.7)	241 (26.4)	
90–99	337 (33.8)	306 (33.6)	
100	69 (6.9)	67 (7.3)	

Abbreviations: EQ = EuroQol; VAS = visual analogue scale.

a Data on economic activity, marital status, religion, ethnicity, day‐to‐day limitations, main language, and responsibility for children unavailable for a minority of participants.

b Data are based on results of the 2011 U.K. Census (Office for National Statistics, 2011), where available; N/A indicates that a directly comparable question was not included in the 2011 Census.

c Data comprises individuals who are separated but still legally married or in a same‐sex civil partnership.

d Data includes individuals who are the surviving partner from a same‐sex civil partnership.

e Census data reported here refers to individuals aged 16–64 only.

### 3.2 Descriptive analysis

**Figure 4:**
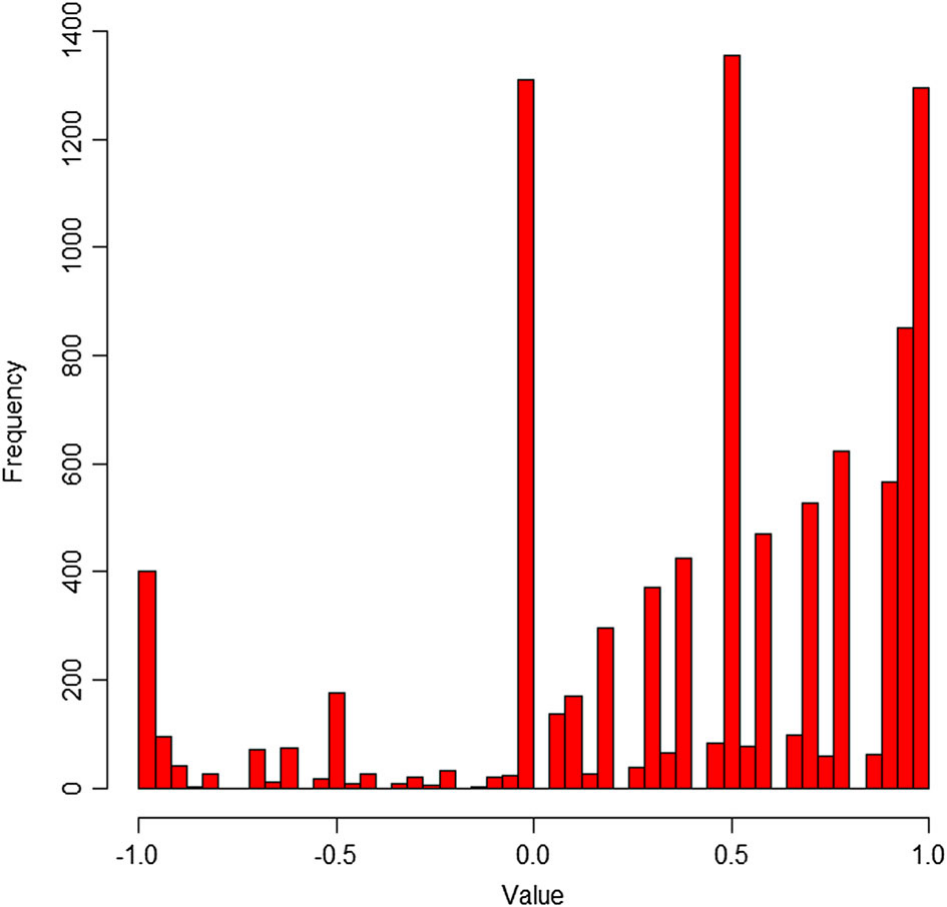
Distribution of observed time trade‐off values [Colour figure can be viewed at wileyonlinelibrary.com]

Figure 4 shows the distribution of observed TTO values. There is some evidence of clustering at key values on the scale (1, 0.5, and 0) and of digit preference (where most values provided correspond to “round” numbers of years in Life A, e.g., 0, 5, and 10 years). Health states were given a value of −1 (indicating that the participants exhausted all of the lead time available to them) on 400 occasions (4.0% of all TTO observations). There are few observations (145; 1.5%) between 0 and −0.5.

We observed evidence of interviewer effects, with different proportions of worse than dead values depending on which interviewer participants were interviewed by.

As a test of the face validity of the data, the means and medians of the TTO values were plotted against the level sum scores of the health states. The results (see Appendix S1) show the expected negative relationship, that is, the worse the health state, the lower the average observed value. Similarly, the proportion of those choosing A or B in the DCE tasks was strongly correlated to the difference in level sum score between the health states, that is, the greater the difference in severity between any two states, the more likely participants were to choose the health state with the lower level sum score.

### 3.3 Exclusion criteria

Twenty‐three participants (2.3%) gave all 10 health states the same value, and 61 participants (6.1%) valued 55555 no lower than the value they gave to the mildest health state. Excluding these participants gave a core modelling dataset of 912 participants (9,120 TTO observations). A review by Engel, Bansback, Bryan, Doyle‐Waters, and Whitehurst (2016) shows that the exclusion of participants who give the same value to all health states or whose data contain logical inconsistencies is common in EQ‐5D valuation studies. Postexclusions, health states were given a value of −1 on 392 occasions (4.3% of all TTO observations).

Of the remaining individuals, 150 participants with more than one health state valued at 0 were treated as censored on the assumption that 0 was the lowest value they were willing to use. Censoring was also applied to 27 participants with inconsistent negative data (where 55555 was given a value of 0 and more than one other health states were given negative values).

### 3.4 Modelling results and the value set

Of the model specifications tested, the 20‐parameter model was selected to generate the value set as it applied no assumptions about the parameters. When addressing the weights given to the different dimensions, the TTO data in the 5‐parameter model suggest weights for mobility, self‐care, usual activities, pain/discomfort, and anxiety/depression of 0.055, 0.043, 0.050, 0.081, and 0.077, respectively. The DCE data in the 5‐parameter model suggest weights of 0.332, 0.238, 0.201, 0.412, and 0.389, respectively. Both methods suggest that pain/discomfort and anxiety/depression receive the greatest weight.

As noted in Section 2.5, we assessed the fit of models with two to 10 latent groups. The DIC statistic improved most when moving from two to three latent groups. The predicted highest and lowest TTO values for the three‐ and 10‐group models are similar, and the three‐group model was chosen to avoid over fitting the data. Our final model is a hybrid model combining the TTO and DCE data and includes three latent groups. The resulting value set is presented in Table 2. Note that in Appendix S2, we present the estimated coefficients for the latent classes, and for the dimensions and levels, separately. However, as the latent class coefficients act to apply an adjustment across all dimensions/level coefficients, Table 2 simplifies the presentation of the value set by reporting the coefficients for dimensions/levels after the application of the latent class coefficients. The minimum value is −0.285 (for the worst health state, 55555) with 5.1% of the 3,125 health states described by the EQ‐5D‐5L being valued as worse than dead. The size of the coefficients in Table 2 reflects the relative weight placed on different sorts of health problems by our sample. For example, at the worst level of problems (Level 5) that can be experienced, pain/discomfort is considered to have the greatest overall impact on HRQL (0.335), followed by anxiety/depression (0.289), mobility (0.274), self‐care (0.203), and usual activities (0.184). At slight levels of problems (Level 2), anxiety/depression has the largest effect on HRQL, followed by pain/discomfort, mobility, self‐care, and usual activities.

**Table 2:** An EQ‐5D‐5L value set for England

	Central estimate a	Value for health state 23245
Constant	1.000	1.000
Mobility		
Slight	0.058	0.058
Moderate	0.076	
Severe	0.207	
Unable	0.274	
Self‐care		
Slight	0.050	
Moderate	0.080	0.080
Severe	0.164	
Unable	0.203	
Usual activities		
Slight	0.050	0.050
Moderate	0.063	
Severe	0.162	
Unable	0.184	
Pain/discomfort		
Slight	0.063	
Moderate	0.084	
Severe	0.276	0.276
Extreme	0.335	
Anxiety/depression		
Slight	0.078	
Moderate	0.104	
Severe	0.285	
Extreme	0.289	0.289
The value for health state 23245	1 − (0.058 + 0.080 + 0.050 + 0.276 + 0.289)	= 0.247

CODA results from final model available from the authors upon request.

a Note that the coefficients reported here are the mean coefficients from the Bayesian regressions.

Table 2 provides a worked example of how to calculate the values for health state 23245, where the relevant decrement for each level of problem on each dimension is subtracted from the constant. The decrements are derived by calculating the weighted averages of the slopes for the three latent groups (not shown; see Appendix S2).

### 3.5 EQ‐5D and EQ‐5D‐5L value set comparisons

**Figure 5:**
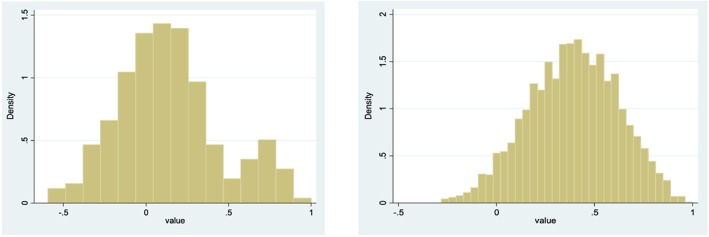
Frequency of values in the EQ‐5D (left) and EQ‐5D‐5L (right) value sets [Colour figure can be viewed at wileyonlinelibrary.com]

Table 3 compares the EQ‐5D‐5L value set with the original EQ‐5D value set (Dolan, 1997) and the crosswalk value algorithm reported by van Hout et al. (2012). The EQ‐5D‐5L value set has a higher value for the worst possible health state and substantially fewer worse than dead values. The decrement from the best (11111) to next best health state is smaller in the EQ‐5D‐5L value set than in the other value sets, as might be expected given differences in number of levels and labelling between the instruments (e.g., 11211 describes “slight” problems performing usual activities in the five‐level instrument and “some” problems in the three‐level version). Pain/discomfort has the largest overall decrement (defined as the change from Levels 1 to 5), whereas usual activities has the smallest. Figure 5a,b shows that the EQ‐5D‐5L value set has a normal distribution, in contrast to the EQ‐5D‐3L value set that was characterised by two peaks (Parkin et al., 2016).

**Table 3:** Comparison of the key characteristics of EQ‐5D‐5L values, crosswalk values, and EQ‐5D‐3L values

	EQ‐5D‐5L value set	Crosswalk value set	EQ‐5D value set
% health states worse than dead	5.1%	26.7%	34.6%
	(159 out of 3,125)	(833 out of 3,125)	(84 out of 243)
Preferences regarding dimensions (ordered from most to least important^b^)	Pain/discomfort	Pain/discomfort	Pain/discomfort
	Anxiety/depression	Mobility	Mobility
	Mobility	Anxiety/depression	Anxiety/depression
	Self‐care	Self‐care	Self‐care
	Usual activities	Usual activities	Usual activities
Value of 55555 (33333)	−0.285	−0.594	−0.594
Value of 11112^a^	0.922	0.879	0.848
Value of 11121^a^	0.937	0.837	0.796
Value of 11211^a^	0.950	0.906	0.883
Value of 12111^a^	0.950	0.846	0.815
Value of 21111^a^	0.942	0.877	0.850
Minimum value	−0.285	−0.594	−0.594
Maximum value	1	1	1

a Note that for each of the asterisked health states, the levels of problems indicated on the five‐level and three‐level versions of EQ‐5D differ, for example, on the EQ‐5D‐5L, 11112 means no problems on any dimension except mild problems with anxiety/depression, whereas on the EQ‐5D, 11112 means no problems on any dimensions except some problems with anxiety/depression. *A priori*, we would expect the values for these health states to be higher in the EQ‐5D‐5L value set than the EQ‐5D value set, which is what we observe.

b Importance is judged by the size of the coefficient for Level 5 in each dimension.

## 4 DISCUSSION

We have reported a value set for the EQ‐5D‐5L, based on the preferences of a random sample of the English general public. Such value sets promote consistency and comparability in assessments of HRQL across different patient groups. The value set can be used to summarise EQ‐5D‐5L data collected from patients in a wide range of contexts and in the economic evaluation of health care interventions to support resource allocation decisions.

The preferences of the English public suggest that pain/discomfort and anxiety/depression are the health problems that are most important, whereas problems with self‐care (ability to wash or dress oneself) and usual activities (e.g., ability to do work, study, housework, family, or leisure activities) are less important. This reflects what members of the public deem important and has implications for the assessment of treatments that affect different aspects of HRQL.

This is one of the first studies internationally to report a value set for the EQ‐5D‐5L. A strength of the study is that the data have been generated using a standard protocol developed following an international programme of work (Oppe et al., 2014). Similar studies are now underway in numerous countries worldwide, which will facilitate direct comparisons of preferences between populations. For example, see Augustovski et al. (2016), Kim et al. (2016), Ramos‐Goñi et al. (2014), Versteegh et al. (2016), and Xie et al. (2016) for details of studies that followed the same protocol. A further strength of our study is that it has addressed problems with previous value sets for EQ‐5D, particularly with respect to the values for worse than dead health states (Devlin et al., 2011; Devlin et al., 2013; Tilling et al., 2010), providing an improved basis for the use of HRQL evidence in decision‐making. Further, innovative methods developed during the course of our study have strengthened the approach to modelling value sets—in particular, by allowing different types of data (TTO and DCE) to be modelled together to provide complementary evidence on preferences, building on earlier work by Oppe and van Hout (2010) and Rowen et al. (2014), and by taking into account the nature of preference data that are “bounded” (censored), heterogeneity of participants' views in health utilities, and heteroskedasticity of the error terms. Further details are provided in Feng et al. (2017). Although our TTO data have face validity, a potential limitation of our study is that there is evidence of clustering at certain values and of selective scale use. This could be linked to the relative difficulty of the TTO task, the different tasks required to value health states better than and worse than dead in the composite TTO, and the use of an automated process to guide its administration. Interviewer effects on TTO responses may also be important given potential differences in levels of interviewer abilities and engagement. We therefore sought to exclude problematic data that could justifiably be considered not to reflect participants' true preferences, whilst avoiding the exclusion of inconvenient data based on subjective researcher judgements. This involved the exclusion of some participants' data and the censoring of some values at and below 0. The alternative approach of including all data would have meant that the assumption of a normal error distribution would need reconsidering. This in turn would have necessitated arbitrary assumptions, potentially with less transparency than the methods we report here. We deemed it invalid to include these data and to knowingly assume the wrong error distribution.

We tested an extensive range of model specifications in our econometric analysis, and each could feasibly have been used to generate a value set. The choice of the model reported in this paper necessarily reflects a number of researcher judgements about which model is “best.” For example, although we could have generated a value set based on TTO data alone, the final value set reported here is derived from a hybrid of both TTO and DCE data, on the grounds that the two methods provide different and complementary information about the views of the sample.

Our choice of the least restrictive (20‐parameter) model is based on two considerations. First, there are strong prior grounds for selecting it, as it allows the values associated with different levels of problems to vary across the dimensions. Second, the overall model statistics, that is, the DIC, suggest that it better captures the nature of the preferences of the English general public than more restrictive models (Feng et al., 2017). Model fit was not improved by including interaction parameters, and so there are no such interaction terms in the value set. This is in contrast to the current EQ‐5D‐3L value set, which included the so‐called “N3 term” (a parameter capturing an additional reduction in value to any health state with a Level 3 problem on any dimension; Dolan, 1997).

The EQ‐5D‐5L value set differs from the original EQ‐5D‐3L value set (Dolan, 1997) and the interim crosswalk EQ‐5D‐5L tariff (van Hout et al., 2012) in important ways. First, the value for the worst state is higher, as expected, given well‐known issues with the procedure for valuing health states worse than dead in the original value set study, which yielded values as low as −39 that required rescaling. As well as a higher minimum value, the value set reported here also has considerably fewer states worse than dead (5.1%, compared to around a third in the original U.K. value set). It is clear that using the new EQ‐5D‐5L value set will mean that some interventions will show larger QALY gains and other interventions will show smaller QALY gains than was previous assumed.

The greater descriptive sensitivity of EQ‐5D‐5L allows patients to give more refined HRQL measurement data as they have more levels over which to describe their health. However, this increased ability to capture responses to treatment may be counteracted by the nature of the value set reported here (Mulhern et al., 2017).

It would be possible to develop a range of value sets, based on the preferences of different population subgroups and methodologies, for use in different contexts. Additional analyses (to be reported separately) show some differences between the health state preferences of different age groups, such that value sets estimated from age‐specific data would differ in important ways. However, the use of a single value set, as mandated by the NICE guidelines (NICE, 2013), allows for consistent decision‐making across patient populations and sociodemographic groups—which is particularly important where resource allocation decisions are concerned. See Sculpher and Gafni (2001) and Robinson and Parkin (2002) for a debate on the use of preference subgroups.

This study raises a range of unanswered questions and areas for further research. First, the value set we have reported is for the English population. However, some health care decisions relate to different jurisdictions. For example, NICE decisions cover both England and Wales. The current U.K. EQ‐5D‐3L value set is used by both NICE and the Scottish Medicines Consortium. Are the preferences of the U.K. population (i.e., including Scotland, Wales, and Northern Ireland) consistent with the values reported here? A U.K. value set drawing on the data reported here for England, combined with additional data collected in Scotland, Wales, and Northern Ireland, will be reported separately.

Similarly, how do the English values compare with those produced in other countries? Over a dozen international EQ‐5D‐5L value set studies using the same protocol as used in this study are either underway or have recently concluded, and future research can compare these in detail.

Finally, although there is evidence to support the face validity of the data used to produce this value set, there are many remaining methodological issues that, if addressed, may help to further improve data quality. For example, changes to the way in which the stated preference tasks and health states are presented to participants may yield improved data. There are also a range of other promising preference elicitation methods that may be used to generate values, such as DCE designs that include an attribute for duration, and can therefore be modelled directly onto the 0 to 1 QALY scale (Bansback, Brazier, Tsuchiya, & Anis, 2012; Mulhern et al., 2014). Although new methods in this research area will continue to be developed, the value set reported here provides a robust and up‐to‐date basis for summarising EQ‐5D‐5L data in decision‐making.

## ETHICAL STATEMENT

The study was reviewed and approved by the Research Ethics Committee at the School of Health and Related Research via the University of Sheffield Ethics Review Procedure.

## ORIGINAL PUBLICATION

This manuscript is not being submitted for publication elsewhere at the same time.

## CONFLICT OF INTEREST

NJD, KKS and YF are employees of the Office of Health Economics, a registered charity which received funding from a variety of sources, including the Association of the British Pharmaceutical Industry. All authors are members of the EuroQol Group.

## Supporting information


**Data S1** Supporting information item
**Appendix I.** The relationship between the means and medians of the TTO values and the level sum scores of the health states
**Appendix II.** An EQ‐5D‐5L value set for England
