# Supplementary material for: Valuing health‐related quality of life: An EQ‐5D‐5L value set for England
Source: Health Econ. 2017 Aug 22;27(1):7–22. doi: 10.1002/hec.3564 (PMC6680214; doi:10.1002/hec.3564)
Supplement: Supplementary file 1 — Data S1 Supporting information item Appendix I. The relationship between the means and medians of the TTO values and the level sum scores of the health states Appendix II. An EQ‐5D‐5L value set for England [file HEC-27-7-s001.zip › Appendix I.docx]

**Appendix I. The relationship between the means and medians of the TTO values and the level sum scores of the health states**
