# Supplementary material for: Valuing health‐related quality of life: An EQ‐5D‐5L value set for England
Source: Health Econ. 2017 Aug 22;27(1):7–22. doi: 10.1002/hec.3564 (PMC6680214; doi:10.1002/hec.3564)
Supplement: Supplementary file 1 — Data S1 Supporting information item Appendix I. The relationship between the means and medians of the TTO values and the level sum scores of the health states Appendix II. An EQ‐5D‐5L value set for England [file HEC-27-7-s001.zip › Appendix II.docx]

**Appendix II. An EQ-5D-5L value set for England**

|  | **Central estimate^i^** | **Standard deviation^ii^** | **Value for health state 23245** |
| --- | --- | --- | --- |
| Constant | 1.000 |  | 1.000 |
| Mobility |  |  |  |
| slight | 0.027 | 0.004 | 0.027 |
| moderate | 0.035 | 0.005 |  |
| severe | 0.096 | 0.006 |  |
| unable | 0.127 | 0.006 |  |
| Self-care |  |  |  |
| slight | 0.023 | 0.004 |  |
| moderate | 0.037 | 0.005 | 0.037 |
| severe | 0.076 | 0.006 |  |
| unable | 0.094 | 0.006 |  |
| Usual activities |  |  |  |
| slight | 0.023 | 0.004 | 0.023 |
| moderate | 0.029 | 0.004 |  |
| severe | 0.075 | 0.005 |  |
| unable | 0.085 | 0.005 |  |
| Pain/discomfort |  |  |  |
| slight | 0.029 | 0.004 |  |
| moderate | 0.039 | 0.005 |  |
| severe | 0.128 | 0.007 | 0.128 |
| extreme | 0.155 | 0.007 |  |
| Anxiety/depression |  |  |  |
| slight | 0.036 | 0.004 |  |
| moderate | 0.048 | 0.005 |  |
| severe | 0.132 | 0.006 |  |
| extreme | 0.134 | 0.006 | 0.134 |
| Probability (group 1) | 0.332 | 0.018 | 0.332x0.992+0.388x2.091+0.281x3.625 |
| Probability (group 2) | 0.388 | 0.020 | =2.159 |
| Probability (group 3) | 0.281 | 0.020 |  |
| Slope (group 1) | 0.992 | 0.031 |  |
| Slope (group 2) | 2.091 | 0.073 |  |
| Slope (group 3) | 3.625 | 0.151 |  |
| The value for health state 23245 | | | 1-2.159x(0.027+0.037+0.023+0.128+0.134)  =0.247 |

^I^ Note that the coefficients reported here are the *mean* coefficients from the Bayesian regressions.

^ii^ Note that standard deviations as reported by WinBugs have most similarity with the concept of standard errors in frequentist statistics.

CODA results from final model available from the authors upon request.
